# Recent advances in conversion therapy for hepatocellular carcinoma with portal vein tumor thrombosis

**DOI:** 10.3389/fonc.2026.1925747

**Published:** 2026-09-30

**Authors:** Panpan Xie, Jianfang Zhu, Peng Xu, Peng Zhang, Fengguo Jian

**Affiliations:** 1 Department of General Surgery, Changyi People’s Hospital, Weifang, China; 2 Department of Hepatobiliary Surgery, Qilu Hospital of Shandong University, Jinan, China

**Keywords:** conversion therapy, hepatocellular carcinoma, immunotherapy, locoregional therapy, perioperative management, portal vein tumor thrombosis, salvage surgery

## Abstract

Portal vein tumor thrombosis (PVTT) is one of the most clinically consequential manifestations of advanced hepatocellular carcinoma (HCC). It reflects aggressive tumor biology, compromises portal venous inflow, and reduces the therapeutic window for locoregional intervention. Historically, macroscopic portal vein invasion generally placed patients within advanced-stage treatment pathways, in which systemic therapy constituted the standard treatment backbone and curative-intent approaches were applicable only to selected patients. This paradigm has now shifted. In recent years, the management of unresectable HCC has been reshaped by immune-based systemic therapy, more active tyrosine kinase inhibitor-based combinations, hepatic arterial infusion chemotherapy (HAIC), selective transarterial therapies, thrombus-directed radiotherapy, and increasingly standardized multidisciplinary conversion strategies. Consequently, a subset of patients with liver-dominant HCC and PVTT can now be downstaged to radical resection or other definitive local treatment, particularly when liver function is preserved and therapy is initiated before irreversible hepatic decompensation develops. This review summarizes recent advances in the diagnosis and classification of PVTT, the biological and technical rationale for conversion therapy, and the evolving evidence for systemic therapy, HAIC, transarterial chemoembolization (TACE), radiotherapy, triple-modality treatment, salvage surgery, and postoperative management. It also highlights key limitations, including the predominance of retrospective East Asian cohorts, heterogeneity in PVTT classification and treatment protocols, uncertainty regarding the optimal timing of surgery, and the absence of validated biomarkers for selecting patients most likely to benefit from aggressive multimodality treatment. Overall, the available evidence supports integrating response-guided multidisciplinary strategies into established evidence-based treatment frameworks for carefully selected patients. Even so, conversion therapy for HCC with PVTT remains an evidence-evolving strategy that should be applied with careful patient selection, disciplined treatment sequencing, and substantial institutional expertise rather than adopted as a universal standard of care.

## 1 Introduction

Hepatocellular carcinoma (HCC) remains one of the deadliest malignancies worldwide and continues to contribute substantially to global cancer mortality (1). Within this already high-risk disease, portal vein tumor thrombosis (PVTT) defines a subgroup with particularly poor prognosis because it facilitates intrahepatic spread, aggravates portal hypertension, compromises effective hepatic perfusion, and narrows the safety margin for both surgery and liver-directed therapy (2, 3). PVTT is therefore more than a radiologic descriptor. It is a major biological and therapeutic turning point that can rapidly transform potentially controllable liver cancer into a condition marked by accelerated progression and short survival when treatment is insufficient or delayed (2).

Historically, most staging systems and treatment algorithms regarded macroscopic vascular invasion as a hallmark of advanced disease (4–6). This logic still strongly influences decision-making in Western practice. The 2024 American Society of Clinical Oncology guideline update continues to place immune-based systemic therapy at the center of first-line treatment for advanced HCC with macrovascular invasion (5), in broad alignment with the European Association for the Study of the Liver (EASL) and Barcelona Clinic Liver Cancer (BCLC) frameworks (4, 6). This recommendation is supported by the SHARP (7), REFLECT (8), IMbrave150 (9), HIMALAYA (10), and HEPATORCH (11) trials, which collectively shifted first-line therapy from sorafenib monotherapy toward more active systemic platforms.

PVTT is not separately codified as a conversion-therapy population in either Western guideline. EASL 2025 places macrovascular invasion within the advanced HCC treatment pathway and retains systemic therapy as the standard treatment backbone for patients who are not candidates for curative or locoregional therapy (4). ASCO 2024 is explicitly a systemic-therapy guideline for advanced HCC. For patients with Child-Pugh A liver function and Eastern Cooperative Oncology Group performance status of 0-1, it strongly recommends atezolizumab plus bevacizumab or durvalumab plus tremelimumab as first-line options (5). Neither guideline provides a PVTT-specific sequence for triple-modality conversion therapy, biomarker-led surgical selection, or routine resection after downstaging. The Western position should therefore not be described as a prohibition of individualized multidisciplinary decisions. Rather, it defines systemic therapy as the guideline-based standard, while intensified locoregional combinations and conversion surgery remain selective, center-dependent strategies outside a routinely recommended PVTT pathway (4, 5).

In parallel, East Asian practice has developed a more differentiated therapeutic philosophy. Chinese and Japanese centers have long emphasized PVTT classification according to the extent of portal involvement, the integration of systemic and locoregional treatment, and the selective use of resection or sequential surgery in carefully chosen patients (3, 12–14). The 2026 Chinese guideline for HCC with PVTT further strengthened this direction by incorporating newer evidence on targeted therapy, immunotherapy, hepatic arterial infusion chemotherapy (HAIC), radiotherapy, transarterial chemoembolization (TACE), and surgery into a classification-based multidisciplinary framework (12).

More specifically, the 2026 Chinese guideline adopts Cheng’s classification as the Chinese standard for PVTT typing and recommends a multidisciplinary treatment decision that integrates liver function, tumor resectability, and PVTT type (12). In resectable Cheng type I or II PVTT, resection is the preferred option. For unresectable disease, the guideline supports individualized combinations of HAIC, TACE, or radiotherapy with targeted therapy and immunotherapy, with conversion therapy and supportive care included as defined components of management (12). This framework does not make surgery a uniform recommendation across PVTT. Instead, it links treatment intensity and therapeutic goals to anatomy, functional reserve, and the possibility of safe radical therapy.

This classification-based framework provides the clinical context for the emergence of conversion therapy. In contemporary HCC practice, conversion therapy refers not simply to tumor shrinkage, but to the deliberate use of systemic and locoregional treatment to convert an initially unresectable tumor into a state in which radical local therapy becomes both feasible and worthwhile (15–17). This concept is particularly relevant to PVTT because monotherapy rarely produces the depth, distribution, and vascular response needed for safe resection (17, 18).

This narrative review synthesizes major guidelines, randomized trials, prospective cohort studies, multicenter retrospective series, and recent meta-analyses available through May 25, 2026, with emphasis on studies directly relevant to PVTT or to conversion-oriented treatment in advanced HCC. Because the evidence base remains heterogeneous and rapidly evolving, the discussion prioritizes clinically practice-shaping data rather than exhaustive study-by-study enumeration (**Figure 1**).

**Figure 1:**
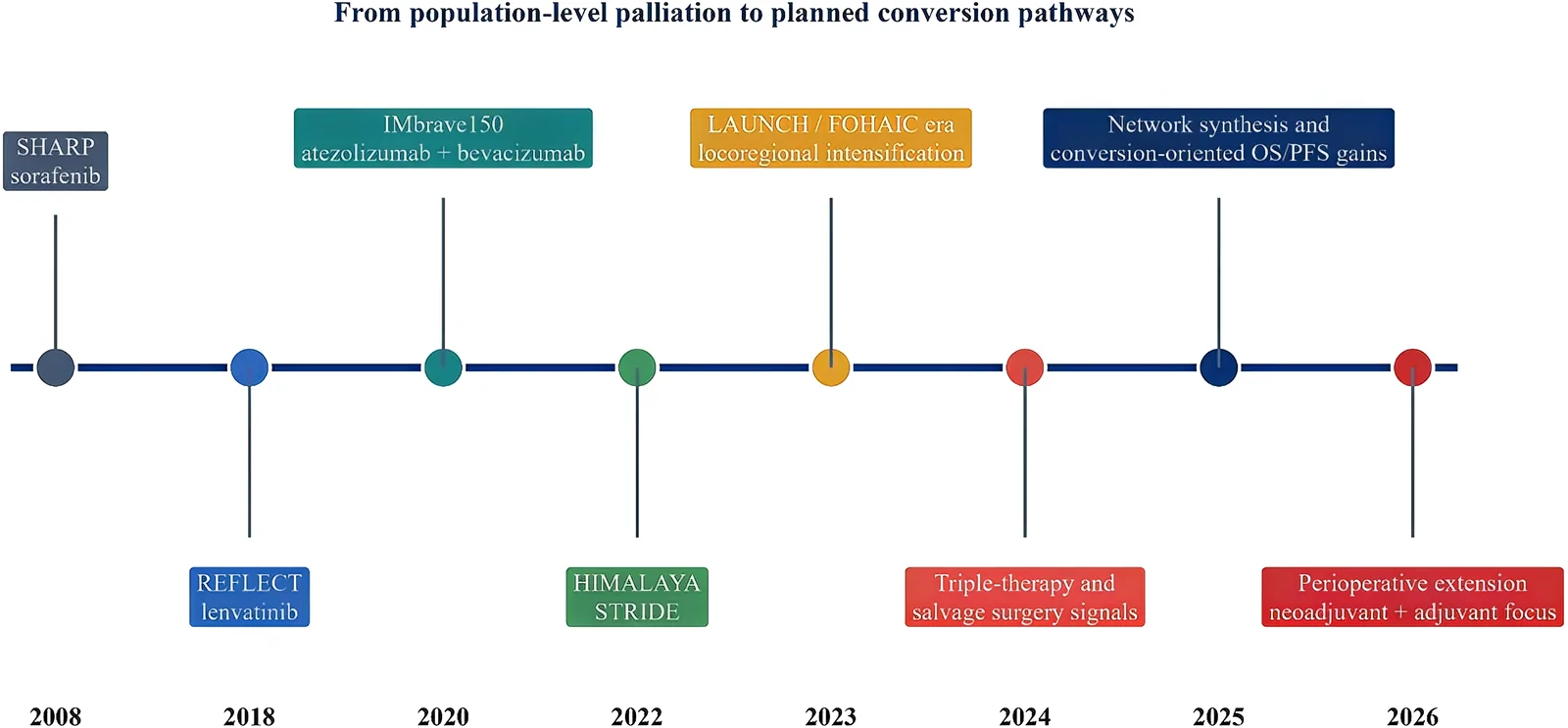
Treatment-evolution timeline for hepatocellular carcinoma (HCC) with portal vein tumor thrombosis (PVTT). This chronological timeline progresses from sorafenib-era monotherapy through immune-based systemic therapy, HAIC intensification, triple-modality treatment, salvage surgery, neoadjuvant strategies, and postoperative targeted-immunotherapy, reflecting the sequential integration of therapeutic modalities over approximately two decades. HAIC, hepatic arterial infusion chemotherapy.

By May 25, 2026, the literature suggested that this pathway was no longer confined to isolated anecdotal success. Combination regimens built around a tyrosine kinase inhibitor, an immune checkpoint inhibitor, and a locoregional modality have produced response and conversion rates that would have been difficult to envision in the sorafenib era (19–21). Emerging data on salvage surgery (22, 23), neoadjuvant strategies for limited PVTT (24–26), and postoperative systemic management in high-risk patients (27, 28) have further extended the conversion concept across the perioperative continuum.

Unlike Lu et al. (14), which broadly reviewed treatment options for HCC with PVTT, and Zhang and Zhang (18), which surveyed conversion therapy across HCC with vascular invasion, this review focuses on a PVTT-specific, classification-based, and response-guided conversion pathway. We incorporate contemporary evidence from the HEPATORCH trial (11), the 2026 Chinese PVTT guideline (12), a 2025 network meta-analysis of triple-modality regimens (19), a 2026 meta-analysis of neoadjuvant treatment (26), and a 2026 multicenter study of postoperative targeted-immunotherapy duration (28). By linking downstaging, neoadjuvant treatment, salvage resection, and adjuvant management, we frame conversion therapy as a perioperative continuum rather than an isolated preoperative intervention. The practical emphasis is on how thrombus anatomy, hepatic reserve, dynamic response, and multidisciplinary capability determine movement between treatment stages and the limits of current evidence.

## 2 Classification and diagnostic evaluation of PVTT

The first diagnostic decision is to determine whether a portal-vein filling defect represents malignant tumor thrombus, bland thrombus, or a mixture of both (29). Tumor thrombus is suggested by enhancing soft tissue within the vein, venous expansion, arterial neovascularity, continuity with the primary tumor, and diffusion restriction, whereas bland thrombus generally lacks enhancement and direct continuity with the tumor and may arise from portal hypertension or thrombosis. This distinction is clinically decisive because the tumor component determines PVTT staging and conversion intensity, while bland thrombus requires evaluation of thrombotic and portal-hypertensive causes and may coexist with malignant thrombus. Multiphasic computed tomography or magnetic resonance imaging, supplemented by contrast-enhanced ultrasound and diffusion-weighted magnetic resonance imaging, can support this distinction (30, 31).

Contrast-enhanced multiphasic computed tomography and magnetic resonance imaging remain the cornerstone of PVTT diagnosis and anatomical assessment (4, 12). These modalities define the level of thrombus extension and clarify the relationship between vascular invasion, intrahepatic tumor distribution, and future remnant liver anatomy. Contrast-enhanced ultrasound can provide an adjunctive assessment when thrombus characterization remains uncertain (30).

A binary description of PVTT as simply present or absent is inadequate for contemporary treatment planning because it obscures major differences between limited branch invasion and extension into the first-order branches or main portal trunk (14, 29). East Asian systems, particularly the Japanese Vp classification and Cheng classification, remain clinically valuable because they connect anatomy with prognosis and treatment strategy (12, 14, 29). The principal classification systems and their implications for conversion planning are summarized in **Table 1** and illustrated in **Figure 2**.

**Table 1:** Portal vein tumor thrombosis (PVTT) classification systems and their relevance to conversion planning.

System	Extent of involvement	Typical clinical implication	Role in conversion planning
Japanese Vp	Vp1–Vp2 branch PVTT; Vp3 first-order branch; Vp4 main trunk/contralateral branch	Greater extent usually predicts poorer prognosis and higher operative complexity	Useful for distinguishing patients suitable for aggressive local therapy from those requiring deeper downstaging
Cheng classification	Type I segmental/sectoral branch; Type II right/left portal vein; Type III main trunk; Type IV superior mesenteric vein	Widely used in Chinese studies and guidelines	Helpful for selecting between TACE-based, HAIC-based, radiotherapy-based, or surgery-oriented strategies

**Figure 2:**
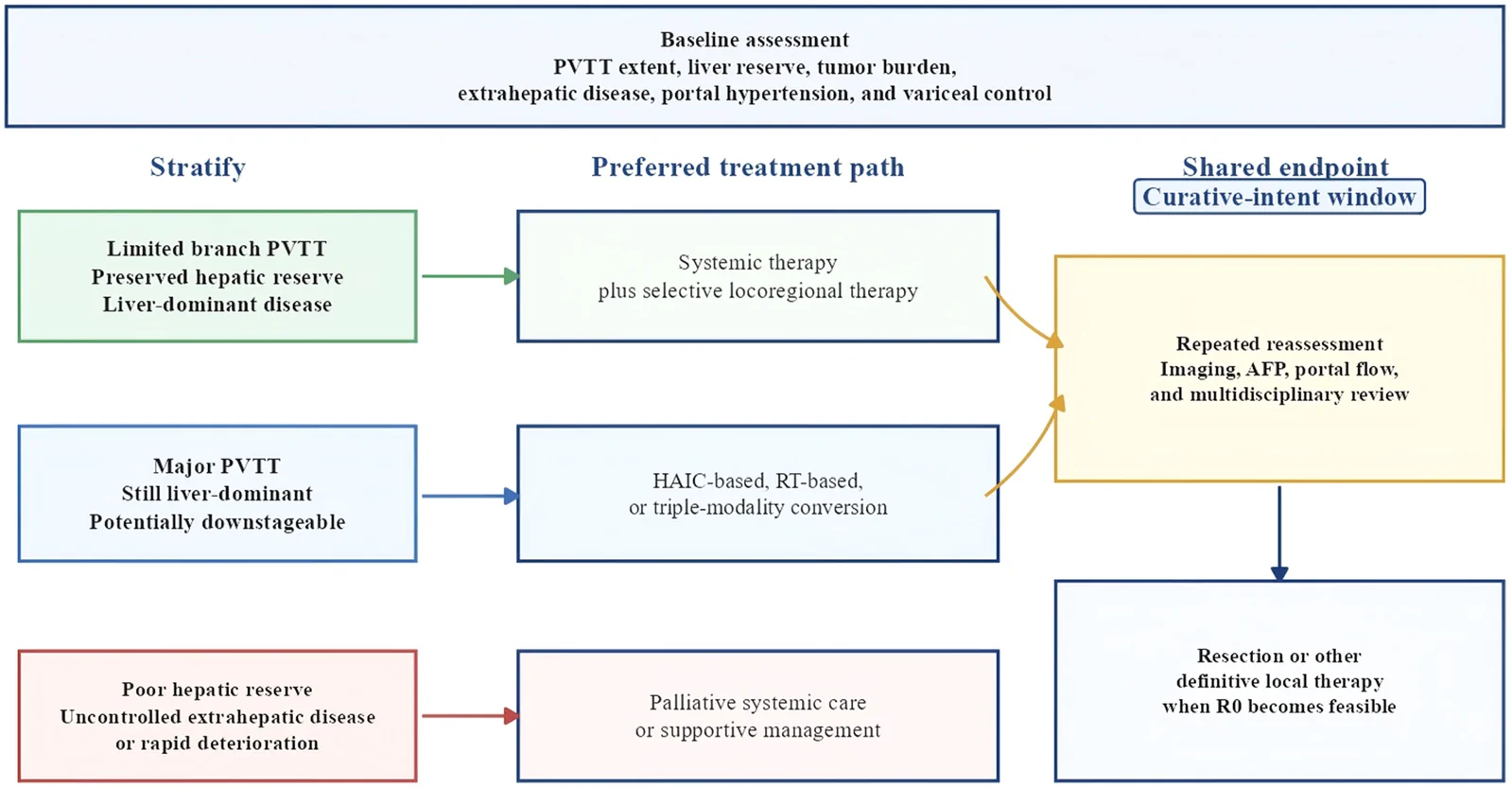
Classification-based conversion pathway for hepatocellular carcinoma (HCC) with portal vein tumor thrombosis (PVTT). This decision algorithm begins with assessment of PVTT extent, liver functional reserve, and extrahepatic disease status, then branches to systemic therapy alone, HAIC-based triple therapy, TACE-based triple therapy, or radiotherapy-based triple therapy according to disease characteristics, and ends with repeated reassessment, surgical resection when feasible, or continued nonoperative management. HAIC, hepatic arterial infusion chemotherapy; TACE, transarterial chemoembolization.

A conversion-oriented evaluation requires more than cross-sectional imaging alone. It should also document Child-Pugh class, albumin-bilirubin grade when available, Eastern Cooperative Oncology Group performance status, the severity of portal hypertension, the presence and control of gastroesophageal varices, alpha-fetoprotein kinetics, future liver remnant sufficiency, and the extent of extrahepatic disease (12, 15, 17). These variables determine whether an intensive multimodal strategy is technically feasible and physiologically defensible.

Staging in the conversion setting is dynamic rather than static. It must be repeatedly updated after each treatment cycle through serial imaging, biomarker trends, liver function testing, and multidisciplinary reassessment (15, 16, 18). Resectability cannot be judged by anatomy alone. A meaningful conversion candidate is one in whom the anticipated pace of tumor and thrombus regression is likely to exceed the pace of hepatic decline.

## 3 Therapeutic rationale for conversion treatment

The rationale for conversion treatment in HCC with PVTT begins with a key distinction: anatomical unresectability and biological unresectability are related, but they are not identical (15, 17, 18). Under effective treatment, portal invasion may regress, a dominant intrahepatic lesion may undergo substantial necrosis, and portal inflow may recover. If liver function remains stable throughout this process, a resection that was initially unsafe or oncologically futile may become achievable.

This concept must be distinguished from simple radiologic response. A patient may achieve a partial response by the Response Evaluation Criteria in Solid Tumors (RECIST) or modified RECIST (mRECIST) and still remain unresectable because thrombus location, tumor multiplicity, or future liver remnant remains unfavorable (22, 23). Conversely, a patient without marked shrinkage of the primary lesion may become operable if the thrombus becomes nonviable or retracts sufficiently to permit a safer hepatectomy.

A 2024 systematic review and meta-analysis of 38 studies involving 4,042 patients with initially unresectable HCC found that the pooled conversion-to-surgery rate was 33% (95% CI, 25-41%) for tyrosine kinase inhibitor (TKI), anti-PD-1, and locoregional therapy, compared with 8% (95% CI, 5-12%) for TKI monotherapy and 28% (95% CI, 19-37%) for TKI plus anti-PD-1 therapy (16). In the lenvatinib, anti-PD-1, and locoregional therapy subgroup, the pooled conversion rate and objective response rate (ORR) were 35% (95% CI, 26-44%) and 70% (95% CI, 56-83%), respectively (16). These pooled estimates are not PVTT-specific, but they quantify why response-intensive regimens are of interest when rapid thrombus control is required. In PVTT, the need for rapid and deep response is even greater because progressive thrombus extension can further compromise portal flow, worsen portal hypertension, and shorten the window for curative-intent intervention (2, 18).

These considerations explain why modern conversion treatment is inherently multidisciplinary. Surgeons evaluate technical resectability and future liver remnant safety. Hepatologists assess liver reserve and complications related to portal hypertension. Medical oncologists optimize systemic disease control. Interventional radiologists and radiation oncologists determine whether intrahepatic or thrombus-directed treatment can be intensified safely (17, 18, 32).

## 4 Advances in systemic therapy

Systemic therapy remains the therapeutic backbone for advanced HCC, including disease complicated by PVTT. In SHARP, sorafenib improved median overall survival (OS) from 7.9 to 10.7 months versus placebo (hazard ratio [HR], 0.69; 95% CI, 0.55-0.87), although the partial response rate was only 2% (7). REFLECT then established lenvatinib as non-inferior to sorafenib for OS (13.6 vs 12.3 months; HR, 0.92; 95% CI, 0.79-1.06) in 954 patients with unresectable HCC (8). IMbrave150 enrolled 501 patients and showed longer median progression-free survival (PFS) with atezolizumab plus bevacizumab than with sorafenib (6.8 vs 4.3 months; HR, 0.59), with an OS HR of 0.58 (9). In HIMALAYA, STRIDE improved median OS to 16.43 months versus 13.77 months with sorafenib (HR, 0.78) (10). HEPATORCH further expanded the East Asian first-line landscape: among 326 patients, toripalimab plus bevacizumab improved median PFS (5.8 vs 4.0 months; HR, 0.69) and median OS (20.0 vs 14.5 months; HR, 0.76) versus sorafenib (11). These pivotal trials establish the systemic efficacy baseline, but were not designed as PVTT-specific conversion studies.

HEPATORCH also has direct safety relevance for PVTT. Randomisation was stratified by macrovascular invasion or extrahepatic spread, but the trial was not designed as a PVTT-specific conversion study (11). Grade 3–4 upper gastrointestinal haemorrhage occurred in 6% of patients receiving toripalimab plus bevacizumab versus 1% receiving sorafenib. Serious upper gastrointestinal haemorrhage occurred in 7% versus 1% (11). For PVTT patients with portal hypertension, these findings support pretreatment assessment and management of high-risk varices, careful selection for bevacizumab, and monitoring for hepatic decompensation (5, 11). HEPATORCH therefore expands the East Asian first-line systemic landscape, but it does not establish conversion efficacy or routine safety for unselected PVTT populations.

These advances are particularly relevant to PVTT because they raise the response baseline from which conversion strategies begin (15, 16). Even so, systemic therapy alone remains insufficient for many patients with extensive PVTT, and the proportion of patients who achieve the anatomical and physiological conditions necessary for curative resection through systemic therapy alone is still limited (2, 18).

Choice of systemic backbone is also constrained by safety and treatment integration. Anti-vascular endothelial growth factor (VEGF)-containing regimens require careful evaluation of bleeding risk, especially in patients with clinically significant portal hypertension or untreated gastroesophageal varices (4, 5). The preferred systemic platform in PVTT must therefore be judged not only by phase 3 efficacy, but also by whether it can be combined safely with HAIC, TACE, radiotherapy, or surgery while preserving liver function.

Geographic practice patterns further influence regimen selection. In China and other parts of East Asia, programmed death-1 (PD-1)-based regimens incorporating agents such as toripalimab, camrelizumab, or sintilimab are commonly used in real-world HCC practice and feature in conversion-oriented protocols (11, 33). Outside East Asia, atezolizumab plus bevacizumab and STRIDE are more commonly used as first-line systemic platforms (4, 5, 9, 10).

## 5 HAIC, TACE, and radiotherapy as conversion partners

### 5.1 Hepatic arterial infusion chemotherapy

Among liver-directed modalities, HAIC has emerged as one of the most important partners for systemic therapy in HCC with PVTT (12, 34, 35). In a randomized phase II trial limited to 64 patients with Vp3 or Vp4 PVTT, sorafenib plus HAIC improved median OS (16.3 vs 6.5 months; HR, 0.28), median PFS (9.0 vs 2.5 months; HR, 0.26), and ORR (41% vs 3%) compared with sorafenib alone (34). Grade 3–4 thrombocytopenia was more frequent with HAIC (22% vs 0%) (34). In the phase III FOHAIC-1 trial (n=262), in which 65.6% of patients had macrovascular invasion, FOLFOX-HAIC improved median OS versus sorafenib (13.9 vs 8.2 months; HR, 0.41); 12.3% of HAIC-treated patients underwent tumor downstaging, although this study was not restricted to PVTT (35).

A 2025 Bayesian network meta-analysis of 10 studies involving 1,241 patients with unresectable HCC and PVTT found that HAIC-based triple therapy was associated with the largest OS benefit versus targeted therapy plus immunotherapy alone (HR, 0.48; 95% CI, 0.32-0.74), followed by radiotherapy-based (HR, 0.53; 95% CI, 0.30-0.91) and TACE-based regimens (HR, 0.65; 95% CI, 0.45-0.94) (19). A 2023 meta-analysis of sorafenib plus HAIC versus sorafenib alone reached a similar conclusion (36).

### 5.2 Transarterial chemoembolization

TACE remains an important but more selectively applied component of the conversion armamentarium (3, 13, 29). In a prospective cohort of 116 patients with HCC and PVTT, propensity score-matched TACE plus lenvatinib was associated with longer median OS (18.97 vs 10.77 months), longer median PFS (10.6 vs 5.4 months), and a higher ORR (66.8% vs 33.3%) than TACE plus sorafenib (37). In the phase III LAUNCH trial of 338 patients with advanced HCC, a population not restricted to PVTT, lenvatinib plus TACE improved median OS (17.8 vs 11.5 months), median PFS (10.6 vs 6.4 months), and ORR by modified RECIST (54.1% vs 25.0%) versus lenvatinib alone (38).

TACE is not a universal solution across the PVTT spectrum. The more proximal the thrombus, the more carefully ischemic risk must be weighed against cytoreductive benefit (13, 29). This is one reason why many centers favor HAIC over TACE in main trunk PVTT while reserving TACE-based strategies for more limited portal involvement.

### 5.3 Radiotherapy

Radiotherapy has moved from a salvage option to a central component of many conversion-oriented regimens. In a randomized trial of 90 treatment-naive patients with liver-confined HCC and macroscopic vascular invasion, TACE plus external beam radiotherapy improved 12-week PFS (86.7% vs 34.3%), 24-week radiologic response (33.3% vs 2.2%), and median OS (55.0 vs 43.0 weeks) compared with sorafenib; five patients (11.1%) underwent resection after downstaging (39). A meta-analysis of radiotherapy modalities for HCC with portal vein thrombosis further suggested that modern conformal techniques can achieve meaningful local control with acceptable toxicity (40).

Radiotherapy may be especially useful when the thrombus itself is the principal barrier to definitive local treatment. This possibility is further supported by randomized data showing that neoadjuvant three-dimensional conformal radiotherapy can improve outcomes in technically resectable patients with limited PVTT (25).

## 6 Triple-modality and multidisciplinary conversion strategies

One of the clearest markers of recent progress is the emergence of triple-modality treatment, typically defined as targeted therapy plus immunotherapy plus one locoregional modality (19, 41). The 2024 Chinese expert consensus on targeted therapy and immunotherapy combined with locoregional therapy formally recognized this approach and provided recommendation-level support for its use in intermediate and advanced HCC, including many patients with PVTT (41). Triple-modality treatment should be embedded in a biomarker-informed, response-guided reassessment pathway rather than treated as a fixed recipe. As summarized in **Figure 3**, radiologic response, PVTT viability, portal flow, liver function, and surgical feasibility remain the primary determinants of conversion. AFP/PIVKA-II kinetics may provide supportive evidence when markers are elevated at baseline, whereas ctDNA, radiomics, and immune profiling remain exploratory adjuncts. Representative studies underpinning this shift are summarized in **Table 2**.

**Figure 3:**
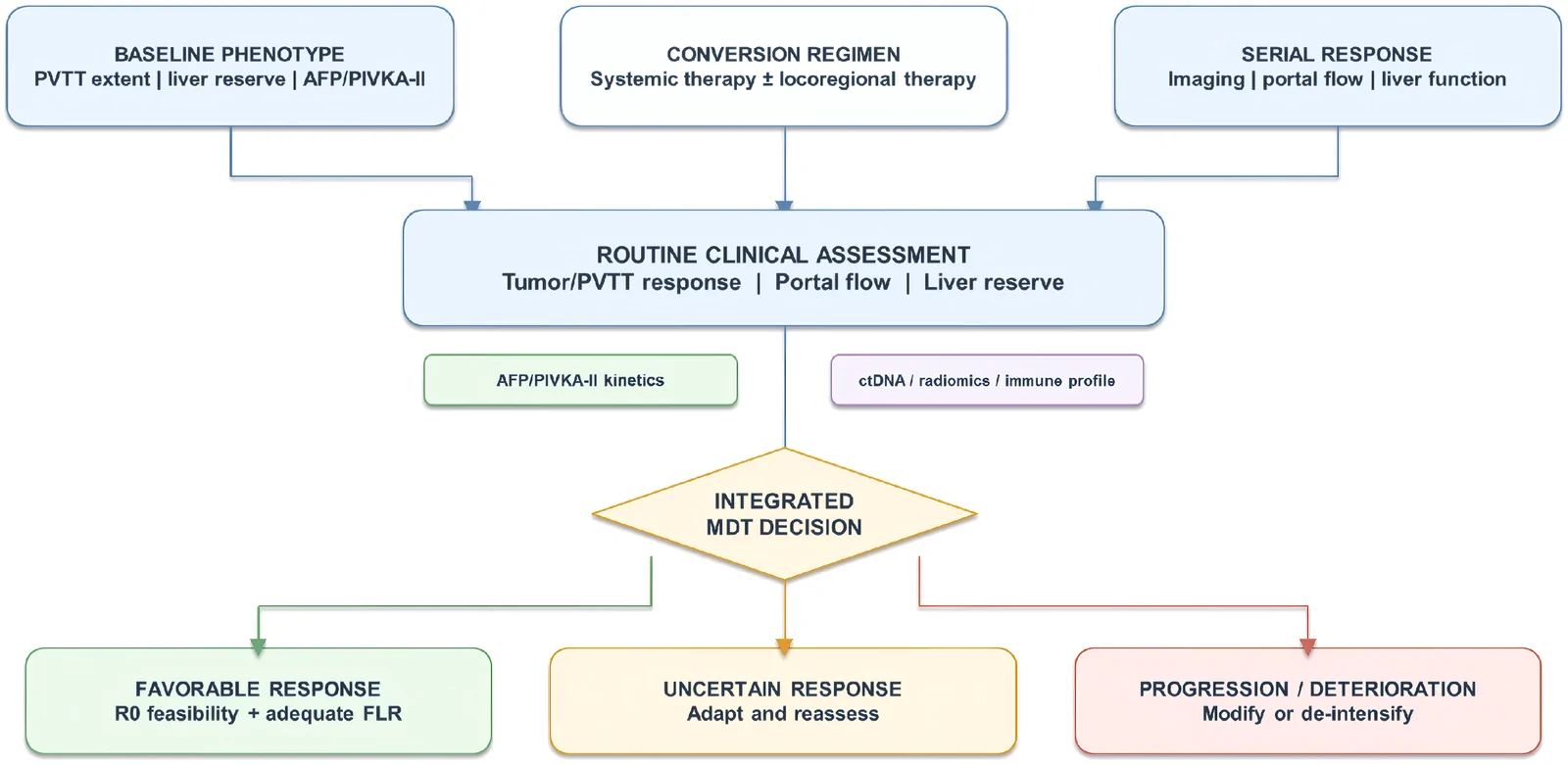
Biomarker-informed pathway for conversion therapy in hepatocellular carcinoma (HCC) with portal vein tumor thrombosis (PVTT). Routine clinical assessment combines contrast-enhanced CT/MRI using mRECIST, PVTT viability, portal flow, liver reserve, and adverse events. AFP/PIVKA-II kinetics may provide supportive evidence when baseline marker elevation permits longitudinal interpretation. ctDNA, radiomics, and immune microenvironment profiling are exploratory adjuncts and should not independently trigger surgery. Surgery is considered only after concordant radiologic response, preserved hepatic reserve, favorable portal inflow, and a technically feasible R0 resection with sufficient future liver remnant. CT, computed tomography; MRI, magnetic resonance imaging; mRECIST, modified Response Evaluation Criteria in Solid Tumors; AFP, alpha-fetoprotein; PIVKA-II, protein induced by vitamin K absence or antagonist-II; ctDNA, circulating tumor DNA; R0, microscopically margin-negative resection.

**Table 2:** Quantitative evidence relevant to conversion-oriented management of hepatocellular carcinoma (HCC) with portal vein tumor thrombosis (PVTT).

Study/design	N	PVTT population/applicability	Regimen (comparator)	ORR/conversion outcome	PFS	OS	Key safety signal	Evidence note
IMbrave150, 2020 (9) Phase III RCT	501–336 vs 165	Unresectable HCC; macrovascular invasion 38%; PVTT-specific outcomes NR	Atezolizumab + bevacizumab vs sorafenib	ORR 27.3% vs 11.9% (RECIST 1.1); conversion NR	6.8 vs 4.3 mo HR 0.59	NR vs 13.2 mo at primary analysis OS HR 0.58	Grade 3–4 AEs: 56.5% vs 55.1%	Systemic benchmark; not a PVTT-specific conversion trial
Sorafenib + HAIC, 2022 (34) Phase II RCT	64–32 vs 32	All major PVTT (Vp3/Vp4)	Sorafenib + 3cir-OFF HAIC vs sorafenib	ORR 41% vs 3%; conversion NR	9.0 vs 2.5 mo HR 0.26	16.3 vs 6.5 mo HR 0.28	Grade 3–4 thrombocytopenia: 22% vs 0%	PVTT-specific randomized evidence; small sample
TACE + lenvatinib, 2021 (37) Prospective cohort with PSM	116–59 vs 57	All HCC with PVTT; Vp/Cheng distribution NR	TACE + lenvatinib vs TACE + sorafenib	ORR 66.8% vs 33.3% after PSM; conversion NR	10.6 vs 5.4 mo after PSM	18.97 vs 10.77 mo after PSM	Reported as safe and well tolerated; detailed Grade 3–4 rates NR	Non-randomized; matched estimates remain susceptible to residual confounding
TACE + external beam RT, 2018 (39) Randomized trial	90–45 vs 45	All liver-confined HCC with macroscopic portal invasion; Child-Pugh A	TACE + external beam RT vs sorafenib	24-wk response: 33.3% vs 2.2%; resection after downstaging: 11.1% vs 0%	12-wk PFS: 86.7% vs 34.3% Median PFS NR	55.0 vs 43.0 wk	No discontinuations for hepatic decompensation in the TACE-RT arm	PVTT-relevant randomized evidence; single tertiary center
LAUNCH, 2023 (38) Phase III RCT	338–170 vs 168	Advanced HCC; PVTT-specific outcomes NR	Lenvatinib + on-demand TACE vs lenvatinib	ORR 54.1% vs 25.0% (mRECIST); conversion NR	10.6 vs 6.4 mo HR 0.43	17.8 vs 11.5 mo HR 0.45	Detailed Grade 3–4 rates NR in primary abstract	All-comer evidence; PVTT was an independent adverse prognostic factor
Lenvatinib + anti-PD-1 + TACE, 2024 (20) Retrospective multicenter	106	All HCC with PVTT	Lenvatinib + anti-PD-1 antibody + TACE	ORR 69.8%; DCR 84.0%; conversion 31.1% (33/106)	12.53 mo	>24 mo	Grade 3–4 TRAEs: 20.8%; no treatment-related deaths	Single-arm retrospective cohort; no contemporaneous control
Lenvatinib + anti-PD-1 + LRT, 2025 (21) Retrospective multicenter	74	All initially unresectable HCC with PVTT	Lenvatinib + anti-PD-1 + TACE, HAIC, or TACE-HAIC	ORR 62.1%; DCR 85.1%; salvage surgery 39.2% (29/74)	13.2 mo 95% CI 8.8-17.6	23.3 mo 95% CI 18.9-27.7	No Grade 5 TRAEs; detailed Grade 3–4 rates NR	Heterogeneous locoregional regimens; selected tertiary-center cohort
Triple-therapy network meta-analysis, 2025 (19) Bayesian network meta-analysis	1,241–10 studies	All studies enrolled unresectable HCC with PVTT	LRT + targeted therapy + immunotherapy vs targeted therapy + immunotherapy	Tumor response analyzed; pooled ORR and conversion rate NR in abstract	Best PFS benefit: RT-based HR 0.43; HAIC-based HR 0.49	Best OS benefit: HAIC HR 0.48; RT HR 0.53; TACE HR 0.65	Grade 3–4 TRAEs: 73.5% vs 39.4%	Predominantly non-randomized constituent studies; regimen heterogeneity
Salvage surgery after TACE + lenvatinib + anti-PD-1, 2023 (22) Retrospective multicenter	70	Initially unresectable HCC; PVTT-specific proportion NR	Triple therapy followed by salvage surgery	All underwent surgery; pCR 41.4%; major pathologic response 84.3%; conversion rate not estimable	Median RFS NR 1-yr 68.9%; 2-yr 54.4%	Median OS NR 1-yr 97.1%; 2-yr 94.4%	Two perioperative deaths; severe postoperative complications 7.1%	Surgery-only cohort; cannot estimate comparative surgical benefit
Salvage surgery after triple conversion, 2024 (23) Retrospective multicenter comparison	93–44 vs 49	All initially unresectable HCC with PVTT after successful conversion	LRT + TKI + anti-PD-1; surgery vs no surgery	No conversion rate reported because successful conversion was an entry criterion	No significant between-group difference P = 0.334	No significant between-group difference P = 0.370	Conversion-treatment AEs comparable; detailed Grade 3–4 rates NR	Retrospective comparison; does not establish a universal survival advantage for surgery
Neoadjuvant 3D conformal RT, 2019 (25) Multicenter RCT	164–82 vs 82	All resectable HCC with PVTT	Neoadjuvant 3D conformal RT + hepatectomy vs hepatectomy	Partial response after RT: 20.7%; all randomized patients planned for surgery	24-mo DFS: 13.3% vs 3.3% Median DFS NR	24-mo OS: 27.4% vs 9.4% Mortality HR 0.35	Detailed Grade 3–4 rates NR in abstract	Randomized perioperative evidence; technically resectable PVTT
Adjuvant TACE + sorafenib, 2024 (27) Phase III RCT	158–79 vs 79	All resected HCC with Cheng type I-III PVTT	Adjuvant TACE + sorafenib vs sorafenib	ORR not applicable in postoperative setting	Median RFS: 16.8 vs 12.6 mo HR 0.57	30.4 vs 22.5 mo HR 0.57	Grade 3–4 hand-foot syndrome: 29.1% vs 30.4%; no deaths	Postoperative, not conversion-treatment evidence
Adjuvant targeted-immunotherapy duration, 2026 (28) Multicenter retrospective cohort	124; groups: 26/28/25/45	Initially unresectable HCC with PVTT after successful conversion and R0 resection	Adjuvant targeted-immunotherapy for 0, 1-3, 4-6, or ?7 months	Not applicable; successful conversion and R0 resection were entry criteria	Vs 0 months: HR 0.44 for 1–3 months and HR 0.15 for 4–6 months; 4–6 vs ?7 months, P = 0.30	Vs 0 months: HR 0.49 for 1–3 months and HR 0.22 for 4–6 months; 4–6 vs ?7 months, P = 0.85	Treatment-related adverse events were manageable and comparable across duration groups	Retrospective duration analysis; susceptible to selection, immortal-time, and treatment-duration confounding

ORR, objective response rate; DCR, disease control rate; PFS, progression-free survival; OS, overall survival; HR, hazard ratio; CI, confidence interval; RCT, randomized controlled trial; NR, not reported; HAIC, hepatic arterial infusion chemotherapy; TACE, transarterial chemoembolization; LRT, locoregional treatment; TKI, tyrosine kinase inhibitor; PD-1, programmed death-1; TRAE, treatment-related adverse event; AE, adverse event; RFS, recurrence-free survival; DFS, disease-free survival; pCR, pathologic complete response.

In a multicenter retrospective study of 106 patients with HCC and PVTT, lenvatinib plus anti-PD-1 antibodies plus TACE produced an ORR of 69.8%, a disease control rate of 84.0%, median PFS of 12.53 months, and median OS exceeding 24 months (20). Conversion to resection occurred in 33 patients (31.1%), while grade 3–4 treatment-related adverse events occurred in 20.8% and no treatment-related deaths were reported (20). In a subsequent multicenter real-world cohort of 74 PVTT patients receiving lenvatinib, anti-PD-1 therapy, and locoregional treatment, ORR was 62.1%, median PFS was 13.2 months, median OS was 23.3 months, and 29 patients proceeded to salvage surgery (21). These estimates are encouraging but hypothesis generating. Both studies were retrospective, lacked a concurrent control group, and were conducted in tertiary East Asian centers. Their populations may have been enriched for patients with preserved liver function, favorable performance status, and access to repeated multidisciplinary assessment. The reported response and conversion rates therefore do not establish comparative benefit or generalizability to unselected PVTT populations.

A 2025 Bayesian network meta-analysis of 10 studies and 1,241 PVTT patients found that the addition of locoregional therapy to targeted therapy plus immunotherapy was associated with improved OS and PFS, with HAIC-based regimens providing the greatest OS benefit (HR, 0.48, 95% CI 0.32-0.74) and radiotherapy-based regimens the greatest PFS benefit (HR, 0.43, 95% CI 0.30-0.63) (19). Grade 3–4 treatment-related adverse events were more common with triple therapy than with targeted therapy plus immunotherapy alone (73.5% vs 39.4%, P < 0.001) (19). Most constituent studies were non-randomized and used different systemic backbones, locoregional techniques, PVTT definitions, and eligibility criteria. The comparative estimates should therefore be interpreted as directional rather than as proof of an optimal triple-modality sequence.

HAIC-based protocols are commonly considered for major PVTT because of their activity in PVTT-focused trials (19, 34). Radiotherapy-based protocols may be considered when thrombus control is the principal technical barrier (25, 39, 40). TACE-based combinations may be considered for limited PVTT with dominant intrahepatic bulk (20, 37, 38). These modality choices remain response-guided and center-dependent. Direct randomized comparisons across contemporary triple-modality regimens are not available.

## 7 Surgery after downstaging and perioperative management

### 7.1 Salvage surgery after successful conversion

The central question in any conversion strategy is whether downstaging can be translated into durable radical treatment. In HCC with PVTT, that endpoint is most often surgical resection (15, 17). Recent surgical series show that selected initially unresectable patients can proceed to hepatectomy after a favorable response to combination treatment (22, 23). Whether surgery itself improves survival beyond continued nonoperative therapy remains uncertain.

A multicenter Liver Cancer study described 70 patients who underwent salvage surgery after TACE, lenvatinib, and anti-PD-1 therapy, with a median interval of 4.3 months from treatment initiation to surgery (22). Pathologic complete response and major pathologic response were observed in 41.4% and 84.3%, respectively. The reported 1- and 2-year OS rates were 97.1% and 94.4%, and corresponding recurrence-free survival rates were 68.9% and 54.4% (22). Because this series included only patients who reached surgery, it cannot estimate the conversion rate, quantify the benefit of surgery over continued therapy, or eliminate selection and guarantee-time bias. A subsequent multicenter comparison of 93 successfully converted PVTT patients found no significant difference in OS or PFS between the 44 patients who underwent salvage surgery and the 49 who did not (23). Current data therefore support surgical feasibility in carefully selected responders, not a universal survival advantage.

### 7.2 Neoadjuvant treatment for technically resectable limited PVTT

A 2024 prospective single-arm study of neoadjuvant-based triple therapy in patients with Cheng type I or II PVTT reported substantial radiologic response and lower microvascular invasion and satellite nodules in resected specimens after a TKI, PD-1 blockade, and TACE (24). The absence of random allocation means that these pathological findings and any apparent survival difference should be regarded as exploratory. Randomized evidence for neoadjuvant three-dimensional conformal radiotherapy is more informative. Among 164 patients with resectable PVTT, 20.7% achieved partial response after radiotherapy, and 24-month OS was 27.4% with neoadjuvant radiotherapy plus hepatectomy versus 9.4% with surgery alone (mortality HR, 0.35) (25).

A 2026 meta-analysis of seven comparative studies involving 621 patients with resectable HCC and PVTT found that neoadjuvant treatment followed by resection was associated with improved OS (HR, 0.48, 95% CI 0.30-0.67) and recurrence-free survival (HR, 0.40, 95% CI 0.20-0.58) versus surgery alone (26). Five of the seven studies were retrospective, one was non-randomized, and only one was randomized. The pooled findings are supportive but remain vulnerable to treatment selection and regional case-mix effects. The randomized radiotherapy data should therefore be interpreted separately (25, 26).

### 7.3 Timing of surgery and postoperative treatment

The 2024 Chinese consensus on sequential surgery after immune-targeted conversion therapy frames surgery as a planned component of the therapeutic continuum rather than as an exceptional last-minute intervention (32). Current practice increasingly relies on serial imaging, alpha-fetoprotein trends, portal flow assessment, performance status, and structured multidisciplinary review rather than on a fixed number of treatment cycles (23, 32). Before surgery is scheduled, serial contrast-enhanced CT or MRI should demonstrate a favorable mRECIST response, with PVTT downstaging or loss of viable enhancement sufficient for an R0 resection. The same reassessment must confirm preserved portal inflow, Child-Pugh A or B function, adequate future liver remnant, ECOG PS 0-1, and no uncontrolled grade 3 or higher treatment-related adverse events. Once technical resectability is achieved, two or more consecutive assessments without further radiologic gain support proceeding to surgery or definitive local therapy rather than continuing treatment to a preset cycle count, while AFP/PIVKA-II decline in marker-positive disease provides supportive but non-decisive evidence (22, 23, 32). A practical framework for selecting candidates for conversion therapy and sequential surgery is provided in **Table 3**.

**Table 3:** Practical framework for selecting candidates for conversion therapy and sequential surgery.

Domain	Preferred features	Red flags	Practical implication
Tumor distribution	Liver-dominant disease; limited extrahepatic spread	Diffuse bilobar progression; uncontrolled extrahepatic disease	Supports whether conversion remains a realistic goal
PVTT anatomy	Classifiable branch or first-order involvement; potentially downstageable main trunk PVTT	Progressive thrombus extension despite treatment	Determines locoregional modality choice and surgical feasibility
Liver reserve	Child-Pugh A, acceptable ALBI, controllable portal hypertension	Borderline hepatic reserve; uncontrolled variceal bleeding risk	Defines whether intensified therapy is physiologically defensible
Dynamic response	AFP decline, thrombus regression, preserved portal flow	Tumor stability with hepatic deterioration	Guides decision to continue downstaging or move to surgery
Institutional capacity	Experienced MDT with hepatobiliary surgery, HAIC/TACE, and radiotherapy expertise	Fragmented care without resectability reassessment	Conversion success depends on coordinated sequencing

PVTT, portal vein tumor thrombosis; ALBI, albumin-bilirubin; AFP, alpha-fetoprotein; MDT, multidisciplinary team; HAIC, hepatic arterial infusion chemotherapy; TACE, transarterial chemoembolization.

A phase III randomized clinical trial of 158 patients with Cheng type I-III PVTT found that adjuvant TACE plus sorafenib improved median recurrence-free survival (16.8 vs 12.6 months; HR, 0.57) and median OS (30.4 vs 22.5 months; HR, 0.57) compared with sorafenib alone after resection (27). Grade 3–4 hand-foot syndrome occurred at similar rates (29.1% vs 30.4%), with no treatment-related deaths (27). In a multicenter retrospective cohort of 124 patients with initially unresectable HCC and PVTT who achieved conversion and subsequently underwent R0 resection, postoperative targeted-immunotherapy duration was associated with both OS and PFS (28). Compared with no adjuvant therapy, the 1-3-month group showed improved OS and PFS (HR, 0.49; P = 0.004 and HR, 0.44; P = 0.010, respectively), while the 4-6-month group showed larger associations with benefit (HR, 0.22; P < 0.001 and HR, 0.15; P < 0.001, respectively) (28). Outcomes did not differ significantly between the 4-6-month and at least 7-month groups for either OS (P = 0.85) or PFS (P = 0.30), and treatment-related adverse events were manageable and comparable across groups (28). Because this study was retrospective, these findings should be considered hypothesis-generating rather than evidence for a universal postoperative treatment duration.

## 8 Biomarkers for response, conversion, and postoperative risk

Biomarkers have three potential roles in conversion therapy for HCC with PVTT. They may identify early biological response, support the timing of definitive local treatment, and estimate postoperative recurrence risk. Their role is complementary. Contrast-enhanced imaging, portal flow, liver reserve, and technical resectability remain the primary determinants of management. No blood, imaging, or tissue biomarker has yet been prospectively validated as a stand-alone selection tool for PVTT-specific conversion therapy. Most evidence is extrapolated from advanced HCC cohorts or from retrospective series conducted at specialized centers. The supporting literature spans liquid biopsy, serum marker kinetics, radiomics, and immune profiling (42–48).

### 8.1 AFP and PIVKA-II kinetics

For patients with marker-positive disease, serial alpha-fetoprotein (AFP) and protein induced by vitamin K absence or antagonist-II (PIVKA-II) measurements offer a low-cost measure of early biological change. In a retrospective cohort of 115 patients receiving radiotherapy-based triple therapy, an AFP decline greater than 50% or a PIVKA-II decline greater than 70% at 6–8 weeks was associated with higher response odds and longer progression-free and overall survival (44). The 2024 Chinese consensus likewise supports serial AFP and PIVKA-II assessment in marker-positive patients as an adjunct to response evaluation (32). These kinetics must be interpreted with imaging. They are not informative in non-secretory tumors, and they can be distorted by hepatic inflammation, biliary obstruction, or treatment-related liver injury.

### 8.2 ctDNA and molecular residual disease

Circulating tumor DNA (ctDNA) may add molecular information that is not visible on cross-sectional imaging. Baseline variant allele fraction may reflect tumor burden. A fall or clearance during therapy may indicate reduced viable disease. Persistent or re-emergent ctDNA after resection may identify molecular residual disease before radiological recurrence. In a prospective study of 41 patients who underwent resection for HCC, ctDNA was detected in 63.4% before surgery and 46.0% after surgery, and serial positivity was associated with shorter recurrence-free survival (45). Analytical sensitivity, low ctDNA shedding, clonal hematopoiesis, assay cost, and the absence of validated decision thresholds currently preclude routine ctDNA-guided conversion or postoperative treatment in PVTT (42, 45).

### 8.3 Imaging and radiomics

Imaging remains central because it defines tumor viability, thrombus extent, portal flow, and future liver remnant. Radiomics may extend this assessment by extracting quantitative features from routine CT or MRI. In a two-center retrospective study of 115 HCC patients with PVTT treated with TACE, lenvatinib, and PD-1 blockade, a pretreatment MRI radiomics model predicted early response with an area under the curve of 0.79 in the test cohort. The combined clinical-radiomics model reached an area under the curve of 0.84 (46). These results are promising, but they require prospective external validation. Feature stability also depends on acquisition protocol, segmentation method, and response definition. Radiomics should therefore support, rather than replace, mRECIST-based multidisciplinary review.

### 8.4 Immune microenvironment profiling

The immune microenvironment may explain why similarly staged tumors respond differently to immune-based conversion regimens. Immune-rich tumors with CD8-positive effector cells and pro-inflammatory macrophage programs appear more likely to benefit from atezolizumab plus bevacizumab, whereas immunosuppressive myeloid populations, including TREM2-positive macrophages, are associated with primary resistance (48). PD-L1 alone has not been established as a clinically actionable selection biomarker in HCC. Tissue sampling also remains challenging in patients with cirrhosis and PVTT. At present, immune profiling is best positioned as a translational endpoint in prospective studies rather than as a prerequisite for routine treatment selection (47, 48).

### 8.5 Clinical integration and current boundary

A practical biomarker strategy should be hierarchical. Serial contrast-enhanced imaging and liver function testing should lead response assessment. AFP and PIVKA-II trends can corroborate a radiological response in marker-positive disease. Discordance between imaging and serology should prompt repeat imaging or multidisciplinary review rather than automatic escalation or surgery. ctDNA, radiomics, and immune profiling are valuable research tools, particularly for prospective trials that define conversion and recurrence endpoints, but current evidence is insufficient to use them as independent triggers for resection or postoperative systemic therapy.

## 9 Current limitations and future directions

Despite the momentum behind conversion therapy, several limitations must be addressed before it can be regarded as a broadly standardized global strategy. Much of the current evidence is derived from retrospective, nonrandomized studies conducted in specialized East Asian centers (3, 12, 14, 15, 18). These cohorts are often dominated by hepatitis B-related disease and supported by mature multidisciplinary infrastructure. They may also preferentially include patients with Child-Pugh A liver function, Eastern Cooperative Oncology Group performance status of 0-1, and liver-dominant disease. Referral patterns, institutional selection for intensified therapy, and the requirement to remain progression-free long enough to undergo surgery can inflate observed conversion and postoperative survival estimates.

Heterogeneity remains pervasive at nearly every level of the literature. PVTT can refer to anything from segmental branch invasion to main trunk or mesenteric extension (12, 14, 29), yet these states are biologically, technically, and prognostically distinct. Triple therapy may involve different tyrosine kinase inhibitors, different PD-1 inhibitors, different HAIC regimens, different embolization strategies, and different radiotherapy techniques (19, 29, 41). Definitions of unresectability, successful conversion, resectability reassessment, and postoperative treatment also vary. Cross-study response, conversion, and survival estimates should not be compared as if they represented a single treatment effect.

Toxicity must remain central to decision-making. Recent evidence synthesis has shown that adding locoregional treatment to targeted therapy plus immunotherapy substantially increases the risk of severe adverse events (19). In patients with cirrhosis, portal hypertension, and borderline hepatic reserve, more treatment is not automatically better. Safety estimates from expert centers may not transfer to settings with different case selection, procedural volume, or supportive care capacity.

The main biomarker limitation is the absence of prospective, PVTT-specific validation. Future studies should predefine biomarker thresholds, use harmonized assays and imaging protocols, and test whether biomarker-informed decisions improve conversion, perioperative safety, or recurrence outcomes.

## 10 Conclusion

The management of HCC with PVTT has changed substantially in recent years. Immune-based first-line systemic therapy has raised the therapeutic baseline. HAIC, TACE, and radiotherapy have each assumed more precise roles as partners in conversion-oriented treatment rather than isolated alternatives. Triple-modality treatment has emerged as the most visible expression of this shift. Salvage surgery, neoadjuvant treatment for limited PVTT, and postoperative targeted-immunotherapy now extend this logic across the perioperative continuum. These developments expand the range of plausible multidisciplinary strategies, but they do not establish a universal treatment sequence.

Even so, caution remains essential. The evidence base is expanding, but it is still uneven, geographically concentrated, and methodologically heterogeneous. Conversion therapy should be considered early in carefully selected patients, but not adopted as a universal standard of care. At present, the most suitable candidates are those with liver-dominant disease, preserved hepatic reserve, anatomically classifiable PVTT, and access to experienced multidisciplinary care. Prospective comparative trials with predefined resectability criteria are needed before broader implementation.

## References

[1] Bray F Laversanne M Sung H Ferlay J Siegel RL Soerjomataram I . Global cancer statistics 2022: GLOBOCAN estimates of incidence and mortality worldwide for 36 cancers in 185 countries. CA Cancer J Clin. (2024) 74:229–63. doi: 10.3322/caac.21834 38572751

[2] Zhou XH Li JR Zheng TH Xu AM . Portal vein tumor thrombosis in hepatocellular carcinoma: molecular mechanism and therapy. Clin Exp Metastasis. (2023) 40:183–97. doi: 10.1007/s10585-022-10188-1 36318440

[3] Chan AWH Zhong J Berhane S Chan SL Kudo M . Management of hepatocellular carcinoma with portal vein tumor thrombosis: Review and update at 2016. World J Gastroenterol. (2016) 22:7289–300. doi: 10.3748/wjg.v22.i32.7289 27621575 PMC4997643

[4] Sangro B Argemi J Ronot M Paradis V Meyer T Mazzaferro V . EASL Clinical Practice Guidelines on the management of hepatocellular carcinoma. J Hepatol. (2025) 82:492–542. doi: 10.1016/j.jhep.2024.08.028 39690085

[5] Gordan JD Kennedy EB Abou Alfa GK Beal E Finn RS Gade TP . Systemic therapy for advanced hepatocellular carcinoma: ASCO guideline update. J Clin Oncol. (2024) 42:940–64. doi: 10.1200/JCO.23.02745 38502889

[6] Reig M Forner A Rimola J Ferrer Fàbrega J Burrel M Garcia Criado A . BCLC strategy for prognosis prediction and treatment recommendation: The 2022 update. J Hepatol. (2022) 76:681–93. doi: 10.1016/j.jhep.2021.11.018 34801630 PMC8866082

[7] Llovet JM Ricci S Mazzaferro V Hilgard P Gane E Blanc JF . Sorafenib in advanced hepatocellular carcinoma. N Engl J Med. (2008) 359:378–90. doi: 10.1056/NEJMoa0708857 18650514

[8] Kudo M Finn RS Qin S Han KH Ikeda K Piscaglia F . Lenvatinib versus sorafenib in first-line treatment of patients with unresectable hepatocellular carcinoma: a randomised phase 3 non-inferiority trial. Lancet. (2018) 391:1163–73. doi: 10.1016/S0140-6736(18)30207-1 29433850

[9] Finn RS Qin S Ikeda M Galle PR Ducreux M Kim TY . Atezolizumab plus bevacizumab in unresectable hepatocellular carcinoma. N Engl J Med. (2020) 382:1894–905. doi: 10.1056/NEJMoa1915745 32402160

[10] Abou Alfa GK Lau G Kudo M Chan SL Kelley RK Furuse J . Tremelimumab plus durvalumab in unresectable hepatocellular carcinoma. NEJM Evid. (2022) 1:EVIDoa2100070. doi: 10.1056/EVIDoa2100070 38319892

[11] Shi Y Han G Zhou J Shi X Jia W Cheng Y . Toripalimab plus bevacizumab versus sorafenib as first-line treatment for advanced hepatocellular carcinoma (HEPATORCH): a randomised, open-label, phase 3 trial. Lancet Gastroenterol Hepatol. (2025) 10:247–59. doi: 10.1016/S2468-1253(25)00059-7 40409323

[12] Chinese Association of Liver Cancer and Chinese Medical Doctor Association . Guidelines for diagnosis and treatment of hepatocellular carcinoma with portal vein tumor thrombus in China (2026 edition). Zhonghua Yi Xue Za Zhi. (2026) 106:41–55. doi: 10.3760/cma.j.cn112137-20251211-03271 41672891. Chinese.

[13] Moriguchi M Furuta M Itoh Y . A review of non-operative treatments for hepatocellular carcinoma with advanced portal vein tumor thrombus. J Clin Transl Hepatol. (2017) 5:177–83. doi: 10.14218/JCTH.2016.00075 28660156 PMC5472939

[14] Lu J Zhang XP Zhong BY Zhang YF Li CQ Sang XT . The progress in the treatment of hepatocellular carcinoma with portal vein tumor thrombus. Front Oncol. (2021) 11:635731. doi: 10.3389/fonc.2021.635731 34631513 PMC8496502

[15] Bai J Huang M Song B Yan Z Yang T . The current status and future prospects for conversion therapy in the treatment of hepatocellular carcinoma. Technol Cancer Res Treat. (2023) 22:15330338231159718. doi: 10.1177/15330338231159718 36855803 PMC9983081

[16] Xu H Zhang H Li B Chen K Wei Y Zou R . Systemic conversion therapies for initially unresectable hepatocellular carcinoma: a systematic review and meta-analysis. BMC Cancer. (2024) 24:1008. doi: 10.1186/s12885-024-12772-y 39143584 PMC11323445

[17] Chinese Expert Panel on Conversion Therapy for Hepatocellular Carcinoma . Chinese expert consensus on conversion therapy for hepatocellular carcinoma (2021 edition). Hepatobiliary Surg Nutr. (2022) 11:227–52. doi: 10.21037/hbsn-21-328 35464283 PMC9023831

[18] Zhang Z Zhang E . Conversion therapy for advanced hepatocellular carcinoma with vascular invasion: a comprehensive review. Front Immunol. (2023) 14:1073531. doi: 10.3389/fimmu.2023.1073531 37180144 PMC10169581

[19] Jiang M Chen C Hu Y Lin G Li H . Locoregional therapy combined with targeted therapy and immunotherapy for hepatocellular carcinoma with portal vein tumor thrombosis: a systematic review and meta-analysis. Sci Rep. (2025) 15:23027. doi: 10.1038/s41598-025-23027-6 41219316 PMC12606199

[20] Ou X Wu J Wu J Fu Y Zeng Z Li S . Efficacy of lenvatinib combined with anti-PD-1 antibodies plus transcatheter arterial chemoembolization for hepatocellular carcinoma with portal vein tumor thrombus: a retrospective, multicenter study. Cancer Res Treat. (2024) 56:1366–79. doi: 10.4143/crt.2023.1165 38697847 PMC11491243

[21] Chi QY Liu QY Wang SJ Liu YD Gao XD Lin KY . Lenvatinib combined with anti-PD-1 antibodies plus locoregional treatment for initial unresectable hepatocellular carcinoma with portal vein tumor thrombosis: a multicenter real-world study. BMC Cancer. (2025) 25:1162. doi: 10.1186/s12885-025-14543-9 40640796 PMC12247254

[22] Wu JY Zhang ZB Zhou JY Ke JP Bai YN Chen YF . Outcomes of salvage surgery for initially unresectable hepatocellular carcinoma converted by transcatheter arterial chemoembolization combined with lenvatinib plus anti-PD-1 antibodies: a multicenter retrospective study. Liver Cancer. (2023) 12:229–44. doi: 10.1159/000528356 37767067 PMC10521320

[23] Wang L Feng JK Lu CD Wu JY Zhou B Wang K . Salvage surgery for initially unresectable HCC with PVTT converted by locoregional treatment plus tyrosine kinase inhibitor and anti-PD-1 antibody. Oncologist. (2024) 29:e1041–50. doi: 10.1093/oncolo/oyae032 38478404 PMC11299929

[24] Hou G Zhang F Feng X Chen Y Zhang J Wang H . Neoadjuvant-based triple therapy for hepatocellular carcinoma with type I/II portal vein tumor thrombosis. J Hepatocell Carcinoma. (2024) 11:1581–95. doi: 10.2147/JHC.S479810 39184154 PMC11344545

[25] Wei X Jiang Y Zhang X Feng S Zhou B Ye X . Neoadjuvant three-dimensional conformal radiotherapy for resectable hepatocellular carcinoma with portal vein tumor thrombus: a randomized, open-label, multicenter controlled study. J Clin Oncol. (2019) 37:2141–51. doi: 10.1200/JCO.18.02184 31283409 PMC6698917

[26] Rajagopal P Vijayagopal KA Kannan SS Shetty S Pai M . Impacts of liver resection with neoadjuvant treatment versus surgery alone in hepatocellular carcinoma with portal vein tumour thrombosis: a systematic review and meta-analysis. Cancers (Basel). (2026) 18:277. doi: 10.3390/cancers18020277 41595196 PMC12838604

[27] Peng Z Fan W Liu Z Xiao H Wu J Tang R . Adjuvant transarterial chemoembolization with sorafenib for portal vein tumor thrombus: a randomized clinical trial. JAMA Surg. (2024) 159:616–25. doi: 10.1001/jamasurg.2024.0506 38568599 PMC10993157

[28] Wang L Feng JK Zhou B Liu ZH Gong L Yan ML . Optimal duration of adjuvant targeted-immunotherapy in patients with initially unresectable hepatocellular carcinoma with portal vein tumor thrombus following successful conversion therapy. Oncologist. (2026) 31:oyag054. doi: 10.1093/oncolo/oyag054 41723827 PMC12995433

[29] Zane KE Makary MS . Locoregional therapies for hepatocellular carcinoma with portal vein tumor thrombosis. Cancers (Basel). (2021) 13:5430. doi: 10.3390/cancers13215430 34771593 PMC8582519

[30] Cerrito L Ainora ME Di Francesco S Galasso L Gasbarrini A Zocco MA . The role of contrast-enhanced ultrasound in the detection of neoplastic portal vein thrombosis in patients with hepatocellular carcinoma. Tomography. (2023) 9:1976–86. doi: 10.3390/tomography9050154 37888746 PMC10610687

[31] Catalano OA Choy G Zhu A Hahn PF Sahani DV . Differentiation of malignant thrombus from bland thrombus of the portal vein in patients with hepatocellular carcinoma: application of diffusion-weighted MR imaging. Radiology. (2010) 254:154–62. doi: 10.1148/radiol.09090304 20032150

[32] Tang H Zhang W Cao J Cao Y Bi X Zhao H . Chinese expert consensus on sequential surgery following conversion therapy based on combination of immune checkpoint inhibitors and antiangiogenic targeted drugs for advanced hepatocellular carcinoma (2024 edition). Biosci Trends. (2024) 18:356–67. doi: 10.5582/bst.2024.01394 39721704

[33] Chen J Hu X Li Q Dai W Cheng X Huang W . Effectiveness and safety of toripalimab, camrelizumab, and sintilimab in a real-world cohort of hepatitis B virus associated hepatocellular carcinoma patients. Ann Transl Med. (2020) 8:1187. doi: 10.21037/atm-20-6063 33241036 PMC7576044

[34] Zheng K Zhu X Fu S Cao G Li WQ Xu L . Sorafenib plus hepatic arterial infusion chemotherapy versus sorafenib for hepatocellular carcinoma with major portal vein tumor thrombosis: a randomized trial. Radiology. (2022) 303:455–64. doi: 10.1148/radiol.211545 35103539

[35] Lyu N Wang X Li JB Lai JF Chen QF Li SL . Arterial chemotherapy of oxaliplatin plus fluorouracil versus sorafenib in advanced hepatocellular carcinoma: a biomolecular exploratory, randomized, phase III trial (FOHAIC-1). J Clin Oncol. (2022) 40:468–80. doi: 10.1200/JCO.21.01963 34905388

[36] Long Y Song X Guan Y Lan R Huang Z Li S . Sorafenib plus hepatic arterial infusion chemotherapy versus sorafenib alone for advanced hepatocellular carcinoma: a systematic review and meta-analysis. J Gastroenterol Hepatol. (2023) 38:292–302. doi: 10.1111/jgh.16088 36516040

[37] Ding X Wang X Ding T Zhang X Yan Z Liu J . TACE plus lenvatinib versus TACE plus sorafenib for unresectable hepatocellular carcinoma with portal vein tumor thrombus: a prospective cohort study. Front Oncol. (2021) 11:821599. doi: 10.3389/fonc.2021.821599 35004336 PMC8733478

[38] Peng Z Fan W Zhu B Wang G Sun J Xiao C . Lenvatinib combined with transarterial chemoembolization as first-line treatment for advanced hepatocellular carcinoma: a phase III, randomized clinical trial (LAUNCH). J Clin Oncol. (2023) 41:117–27. doi: 10.1200/JCO.22.00392 35921605

[39] Yoon SM Ryoo BY Lee SJ Kim JH Shin JH An JH . Efficacy and safety of transarterial chemoembolization plus external beam radiotherapy vs sorafenib in hepatocellular carcinoma with macroscopic vascular invasion: a randomized clinical trial. JAMA Oncol. (2018) 4:661–9. doi: 10.1001/jamaoncol.2017.5847 29543938 PMC5885246

[40] Rim CH Kim CY Yang DS Yoon WS . Comparison of radiation therapy modalities for hepatocellular carcinoma with portal vein thrombosis: a meta-analysis and systematic review. Radiother Oncol. (2018) 129:112–22. doi: 10.1016/j.radonc.2017.11.013 29233562

[41] Bi X Lu Y Chen B Yang Z Hong Z Wang H . Chinese expert consensus on the combination of targeted therapy and immunotherapy with locoregional therapy for intermediate/advanced hepatocellular carcinoma. Liver Cancer. (2024) 13:640–57. doi: 10.1159/000540857 40547962 PMC12180795

[42] Chidambaranathan-Reghupaty S Fisher PB Sarkar D . Circulating tumor DNA clinical applications in hepatocellular carcinoma: current trends and future perspectives. Clin Chem. (2024) 70:33–47. doi: 10.1093/clinchem/hvad168 37962158

[43] Espejo Cruz ML González Rubio S Zamora Olaya J Amado Torres V Alejandre R Sánchez Frías M . Circulating tumor cells in hepatocellular carcinoma: a comprehensive review and critical appraisal. Int J Mol Sci. (2021) 22:13073. doi: 10.3390/ijms222313073 34884878 PMC8657934

[44] Zhang T Li W Chen Q He W Sun J Li D . Prognostic significance of early alpha fetoprotein and des-gamma carboxy prothrombin responses in unresectable hepatocellular carcinoma patients undergoing triple combination therapy. Front Immunol. (2024) 15:1508028. doi: 10.3389/fimmu.2024.1508028 39726604 PMC11669689

[45] Zhu GQ Liu WR Tang Z Qu WF Fang Y Jiang XF . Serial circulating tumor DNA to predict early recurrence in patients with hepatocellular carcinoma: a prospective study. Mol Oncol. (2022) 16:549–61. doi: 10.1002/1878-0261.13105 34543520 PMC8763657

[46] Lu D Zhou L Zuo Z Zhang Z Zheng X Weng J . MRI radiomics to predict early treatment response to TACE combined with lenvatinib plus a PD-1 inhibitor for hepatocellular carcinoma with portal vein tumor thrombus. J Hepatocell Carcinoma. (2025) 12:985–98. doi: 10.2147/JHC.S513696 40406667 PMC12094907

[47] Donne R Lujambio A . The liver cancer immune microenvironment: therapeutic implications for hepatocellular carcinoma. Hepatology. (2023) 77:1773–96. doi: 10.1002/hep.32740 35989535 PMC9941399

[48] Cappuyns S Piqué Gili M Esteban Fabró R Philips G Balaseviciute U Pinyol R . Single-cell RNA sequencing-derived signatures define response patterns to atezolizumab plus bevacizumab in advanced hepatocellular carcinoma. J Hepatol. (2025) 82:1036–49. doi: 10.1016/j.jhep.2024.12.016 39709141 PMC12086051

